# Clinical characteristics of non‐small cell lung cancer patients with *EGFR* mutations and *ALK&ROS1* fusions

**DOI:** 10.1111/crj.13472

**Published:** 2022-01-26

**Authors:** Qinghua Liu, Qingyan Huang, Zhikang Yu, Heming Wu

**Affiliations:** ^1^ Center for Pathological Diagnostics, Meizhou People's Hospital (Huangtang Hospital) Meizhou Academy of Medical Sciences Meizhou P. R. China; ^2^ Guangdong Provincial Key Laboratory of Precision Medicine and Clinical Translational Research of Hakka Population, Meizhou People's Hospital (Huangtang Hospital) Meizhou Academy of Medical Sciences Meizhou P. R. China; ^3^ Center for Precision Medicine, Meizhou People's Hospital (Huangtang Hospital) Meizhou Academy of Medical Sciences Meizhou P. R. China

**Keywords:** *ALK*, clinical characteristics, *EGFR*, emphysema, *ROS1*

## Abstract

**Objective:**

To study the relationship between clinical characteristics and anaplastic lymphoma kinase (*ALK*) fusions, c‐ros oncogene 1, receptor tyrosine kinase (*ROS1*) gene fusions, and epidermic growth factor receptor (*EGFR*) mutations in non‐small cell lung cancer (NSCLC) patients to distinguish these different types.

**Methods:**

Both *ALK*, *ROS1* gene rearrangements and *EGFR* mutations testing were performed. The clinical characteristics and associated pulmonary abnormalities were investigated.

**Results:**

Four hundred fifty‐three NSCLC patients were included for analysis. One hundred seventy (37.5%), 32 (7.1%), and 9 cases (2.0%) with *EGFR* mutations, *ALK* gene fusions, and *ROS1* gene fusions were identified, respectively. The *EGFR*‐positive and *ALK&ROS1*‐positive were more common in female (*χ* ^2^ = 61.934, *P* < 0.001 and *χ* ^2^ = 28.152, *P* < 0.001), non‐smoking (*χ* ^2^ = 59.315, *P* < 0.001 and *χ* ^2^ = 11.080, *P* = 0.001), and adenocarcinoma (*χ* ^2^ = 44.864, *P* < 0.001 and *χ* ^2^ = 12.318, *P* = 0.002) patients; proportion of patients with emphysema was lower (*χ* ^2^ = 35.494, *P* < 0.001 and *χ* ^2^ = 15.770, *P* < 0.001) than the wild‐type patients. The results of logistic regression analysis indicated that female (adjusted odds ratio [OR] 1.834, 95% confidence interval [CI] 1.069–3.144, *P* = 0.028), non‐smoking (adjusted OR 2.504, 95% CI 1.456–4.306, *P* = 0.001), lung adenocarcinoma (adjusted OR 4.512, 95% CI 2.465–8.260, *P* < 0.001), stage III–IV (adjusted OR 2.232, 95% CI 1.066–4.676, *P* = 0.033), and no symptoms of emphysema (adjusted OR 2.139, 95% CI 1.221–3.747, *P* = 0.008) were independent variables associated with *EGFR* mutations. Young (adjusted OR 3.947, 95% CI 1.873–8.314, *P* < 0.001) and lung adenocarcinoma (adjusted OR 2.950, 95% CI 0.998–8.719, *P* = 0.050) were associated with *ALK/ROS1* fusions.

**Conclusions:**

*EGFR* mutations were more likely to occur in non‐smoking, stage III–IV, and female patients with lung adenocarcinoma, whereas *ALK&ROS1* gene fusions were more likely to occur in young patients with lung adenocarcinoma. Emphysema was less common in patients with *EGFR* mutations.

**Abbreviations**
ALK: anaplastic lymphoma kinase
ARMS: amplification refractory mutation system
EGFR: epidermic growth factor receptor
EML4: echinoderm microtubule associated protein‐like 4
NSCLC: non‐small cell lung cancer
ROS1: proto‐oncogene protein tyrosine kinase ROS

## 1 INTRODUCTION

With the increasing morbidity and mortality, lung cancer has become the first malignant tumor. Lung cancer has the highest morbidity and mortality among men in China.^1^, ^2^, ^3^, ^4^ The morbidity of lung cancer among women in China ranks second and the mortality rate of lung cancer among women is the first.^5^ Non‐small cell lung cancer (NSCLC) accounts for more than 80% of all patients with lung cancer.^6^, ^7^

In recent years, personalized molecular targeted therapy as the core has become a research hotspot in the treatment of lung cancer.^8^, ^9^, ^10^ Currently, the most commonly used treatment of NSCLC is molecular targeted drugs targeting at mutations of epidermic growth factor receptor (*EGFR*), also known as small‐molecule tyrosine kinase inhibitors (TKIs), which have obvious clinical efficacy on NSCLC patients with *EGFR* sensitive mutations.^11^ EGFR is a transmembrane tyrosine kinase receptor, expressed in a variety of epithelial tumors, and regulates tumor cell growth, invasion, transformation, angiogenesis, and metastasis by activating downstream signal transduction proteins.^12^ The main mechanism of EGFR‐TKI is to selectively bind ATP binding sites in the tyrosine kinase domain of EGFR in cells, block the phosphorization and activation of tyrosine itself in EGFR molecules through the Akt‐MAPK pathway, and inhibit RAS/RAF/MAPK, PI3K‐Akt, and other downstream signaling pathways and lead to apoptosis of tumor cells.^13^

Echinoderm microtubule associated protein‐like 4 (*EML4*) and anaplastic lymphoma kinase (*ALK*) fusion gene *EML4‐ALK* and proto‐oncogene protein tyrosine kinase ROS (*ROS1*) were found after *EGFR* mutations in NSCLC.^14^, ^15^, ^16^ *ALK* and *ROS1* rearrangements define important molecular subgroups of NSCLC. After discovery of *ALK* rearrangements in NSCLC, it was recognized that these confer sensitivity to ALK inhibition.^17^ For NSCLC patients with *ALK* gene fusion and *ROS1* gene fusion, targeted therapy with Crizotinib could achieve better efficacy.^18^ Therefore, the detection of *EGFR*, *ALK*, and *ROS1 (ALK&ROS1)* gene mutations before targeted therapy is of great significance for the prediction of the efficacy of targeted therapy and the appropriate patient screening.

The mutation status of targeted therapy driver gene in NSCLC is closely related to its pathological classification. The differences between different pathological subtypes of lung cancer are of great significance for clinical treatment and prognosis of lung cancer patients. The relationship between the mutation status of targeted therapeutically driven genes and clinicopathology in NSCLC has not been consistent. The purpose of this study was to evaluate the clinical characteristics of NSCLC patients in order to distinguish *ALK&ROS1* gene rearrangements, *EGFR* mutations, and non‐*ALK&ROS1/EGFR* (no mutations and rearrangements), so as to distinguish these different types, to assist clinicians to assess the NSCLC patients with these genetic abnormalities.

## 2 MATERIALS AND METHODS

### 2.1 Specimen collection

Test specimens of NSCLC patients were collected from the Meizhou People's Hospital (Huangtang Hospital) (Meizhou, Guangdong, China) between April 2018 and August 2019. All protocols were approved by the Human Ethics Committees of Meizhou People's Hospital, Meizhou Academy of Medical Sciences. The medical records of each patient were reviewed and the corresponding clinical characteristics were extracted.

### 2.2 DNA and RNA extraction

Ten pieces of formalin‐fixed and paraffin‐embedded (FFPE) slices (5 μm thick per slice) were placed into a 1.5‐ml EP tube. After FFPE slices were deparaffinized, DNA and RNA were extracted by AmoyDx® Tissue DNA/RNA Co‐separation Kit (Spin Column) (Amoy Diagnostics, Xiamen, China), following the manufacturers' instructions, and the quantity and quality of extracted DNA and RNA were evaluated.

### 2.3 Detection of *EGFR* gene mutations by ARMS PCR

*EGFR* gene mutations were detected by real‐time amplification refractory mutation system (ARMS)‐PCR, and the 29 mutational hotspots from exon 18 to 21 in this gene were covered with the *EGFR* Gene Mutations Fluorescence Polymerase Chain Reaction Diagnostic Kit (Amoy Diagnostics, Xiamen, China). PCR was performed with initial denaturation at 95°C for 5 min, followed by 15 cycles of first amplification (at 95°C for 25 s, 64°C for 20 s, and 72°C for 20 s) and 31 cycles of second amplification (at 95°C for 25 s, 60°C for 35 s, and 72°C for 20 s). Positive results were defined as Ct (sample) − Ct (control) < Ct (cut‐off) according to the criteria defined by the manufacturer's instructions.

### 2.4 Detection of *ALK* and *ROS1* gene fusions by RT‐PCR

The fusion genes of *ALK* and *ROS1* were analyzed by RT‐PCR according to the manufacturer's protocol of AmoyDx® *ALK* Gene Fusions and *ROS1* Gene Fusions Detection Kit (Amoy Diagnostics, Xiamen, China) with the LightCycler 480 real‐time PCR system. The detection range included the fusions of *ALK* gene with *EML4*, *KIF5B*, *TFG*, and *KLC1* genes, and the fusions of *ROS1* gene with *SLC34A2*, *CD74*, *SDC4*, *EZR*, *TPM3*, *LRIG3*, and *GOPC* genes. Reverse transcription reaction system: reverse transcriptase 0.5 μl, RNA template 6 μl (total RNA 0.5–5.0 μg), 42°C for 1 h, and 95°C for 5 min. PCR amplification: 1.5 μl *ALK&ROS1* mixed enzyme was respectively taken to *ALK* cDNA and *ROS1* cDNA of the samples to be tested, 5 μl was successively transferred to the eight‐tube strip PCR reaction system, and negative and positive controls were set up. The detection instrument and circulating conditions were the same as *EGFR* mutation detection, and Ct values were also interpreted.

### 2.5 Statistical analysis

All analysis was conducted using SPSS statistical software Version 21.0. Fisher's exact test and the Student's *t*‐test were performed in this study. *EGFR*‐positive group, *ALK&ROS1*‐positive group, and non‐*ALK&ROS1/EGFR* group, pairwise comparisons were performed. The relationship between *EGFR*, *ALK*, and *ROS1* genes mutations and clinical characteristics, various types of mutations in the *EGFR* gene, and clinical characteristics were analyzed. Logistic regression analysis was applied to assess the variables independently associated with EGFR and ALK/ROS1 genes mutations. *P* < 0.05 is considered statistically significant.

## 3 RESULTS

### 3.1 Population characteristics

**FIGURE 1:**
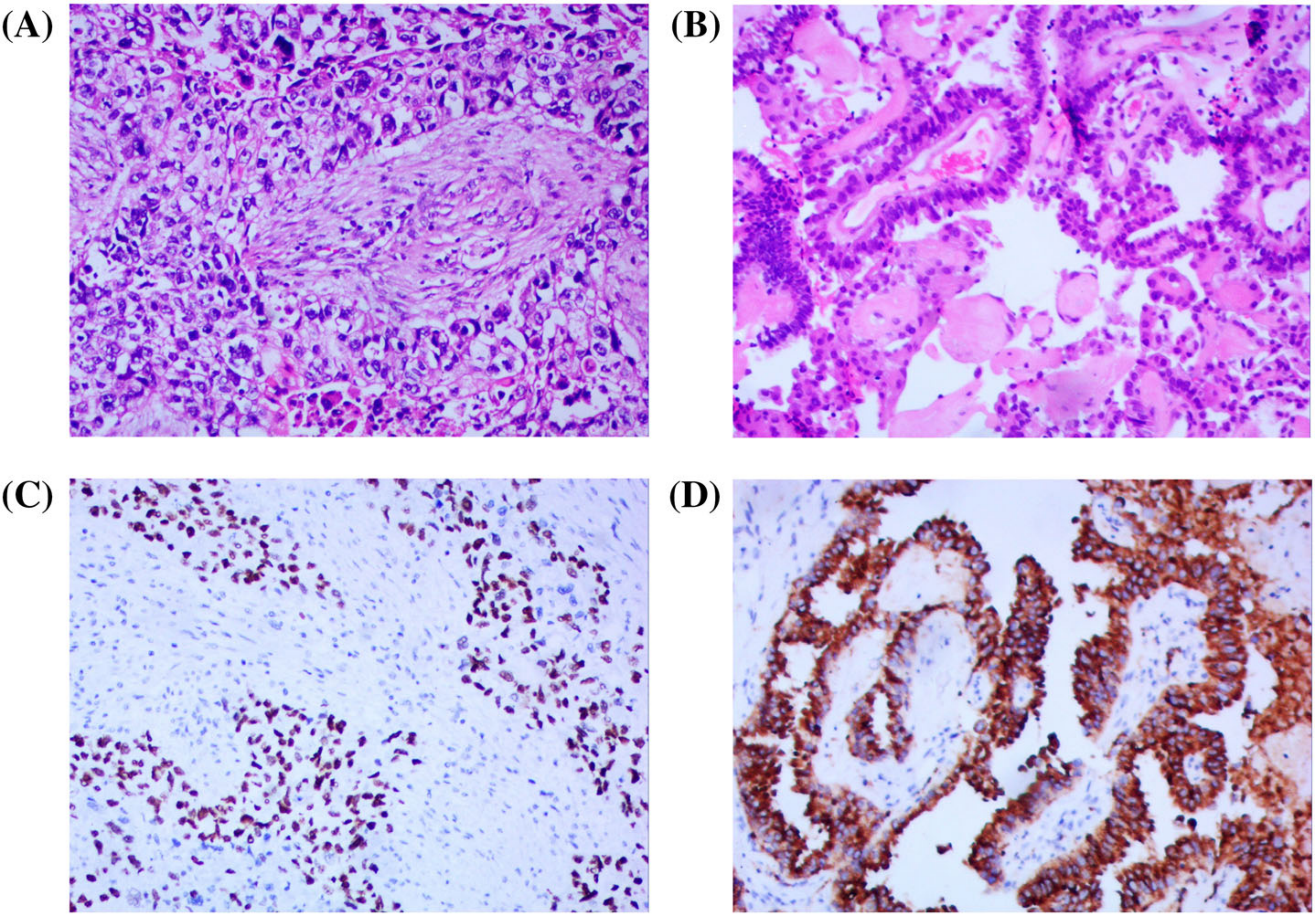
Pathological features of non‐small cell lung cancer. (A) Hematoxylin–eosin (HE) staining of squamous cell carcinoma; (B) HE staining of adenocarcinoma; (C) immunohistochemical staining of P63 expression in squamous cell carcinoma; (D) immunohistochemical staining of Napsin A expression in adenocarcinoma; scale bar, 100 μm

This study involved 453 Chinese NSCLC patients who performed with *EGFR* mutation and *ALK&ROS1* fusion test. A total of 308 patients were male and 145 were female. There were 341 (341/453, 75.3%) patients with lung adenocarcinoma, 106 (106/453, 23.4%) patients with lung squamous cell carcinoma, and 6 (6/453, 1.3%) patients with lung adenosquamous cell carcinoma, respectively. Hematoxylin–eosin (HE) staining and immunohistochemical staining for squamous cell carcinoma and lung adenocarcinoma are shown in Figure 1. Most of these patients, 248 (248/453, 54.7%) were nonsmokers and 269 (269/453, 59.4%) were older than 60 years old. Thirty‐three (7.3%), 17 (3.8%), 90 (19.9%), and 313 (69.1%) patients were in stage I, II, III, and IV, respectively. The clinical characteristics of patients are shown in Table 1.

**TABLE 1:** Characteristics of patients with lung cancer included in this study

Parameter	*n* (%)
Age (years)	
≤60	184 (40.6)
>60	269 (59.4)
Mean ± SD	61.92 ± 10.24
Range	27–87
Gender	
Male	308 (68.0)
Female	145 (32.0)
Smoking status	
No smoking	248 (54.7)
Smoking	205 (45.3)
Pathology	
Adenocarcinoma	341 (75.3)
Squamous	106 (23.4)
Adenosquamous	6 (1.3)
Disease stage	
I	33 (7.3)
II	17 (3.7)
III	90 (19.9)
IV	313 (69.1)

### 3.2 Comparisons of characteristics between *EGFR*‐positive and *ALK&ROS1*‐positive cases, *ALK&ROS1*‐positive and non‐*ALK&ROS1/EGFR* cases, and *EGFR*‐positive and non‐*ALK&ROS1/EGFR* cases in NSCLC patients

A total of 170 cases with *EGFR* mutations in exons 18, 19, 20, or 21 (170/453, 37.5%) were identified in the 453 patients. The G719X mutation (in exon 18) was identified in 1 case (0.6%); exon 19 deletion were identified in 90 cases (57.0%), exon 20 insertion were detected in 2 cases (1.3%), L858R mutation (in exon 21) were detected in 60 cases (38.0%), and L861Q mutation (in exon 21) were detected in 5 cases (3.2%). *ALK* gene fusions were identified in 32 cases (32/453, 7.1%) and *ROS1* gene fusions were identified in 9 cases (9/453, 2.0%).

Compared with the *EGFR*‐positive group, the significant differences in *ALK&ROS1*‐positive group were younger (*P* < 0.001). There were no significant differences in gender, smoking history, histologic type, clinical stage, and computed tomography characteristics (lymphangitis, lymphadenopathy, emphysema, fibrosis, and pleural effusion).

The characteristics of patients with *EGFR*‐positive and non‐*ALK&ROS1/EGFR* (wild type) were compared. In the *EGFR*‐positive group, the majority were female (*χ* ^2^ = 61.934, *P* < 0.001), non‐smoking (*χ* ^2^ = 59.315, *P* < 0.001), and adenocarcinoma (*χ* ^2^ = 44.864, *P* < 0.001) patients. In addition, there were significant differences in clinical stage (*χ* ^2^ = 14.642, *P* = 0.002), proportion of patients with emphysema (*χ* ^2^ = 35.494, *P* < 0.001), and pulmonary fibrosis (*χ* ^2^ = 4.529, *P* = 0.038). There were no significant differences in age, lymphangitis, lymphadenopathy, and pleural effusion.

Compared with the non‐*ALK&ROS1/EGFR* cases in NSCLC patients, the *ALK&ROS1*‐positive group that significantly differed from the non‐*ALK&ROS1/EGFR* group was younger (*χ* ^2^ = 19.920, *P* < 0.001); the majority were female (*χ* ^2^ = 28.152, *P* < 0.001), non‐smoking (*χ* ^2^ = 11.080, *P* = 0.001), and adenocarcinoma (*χ* ^2^ = 12.318, *P* = 0.002) patients; proportion of patients with lymphangitis was higher (*χ* ^2^ = 4.647, *P* = 0.046); and proportion of patients with emphysema was lower (*χ* ^2^ = 15.770, *P* < 0.001) (Table 2).

**TABLE 2:** Comparisons of characteristics between *EGFR*‐positive and *ALK/ROS1*‐positive cases, *ALK/ROS1*‐positive and non‐*ALK&ROS1/EGFR* cases, and *EGFR*‐positive and non‐*ALK/ROS1/EGFR* cases in non‐small cell lung cancer patients

Patient characteristic	*EGFR* (+) (*n* = 170)	*ALK/ROS1* fusion (+) (*n* = 41)	Non‐*ALK/ROS1/EGFR* (wild type) (*n* = 244)	*P* value		
				*EGFR* (+) versus *ALK/ROS1* fusion (+)	*EGFR* (+) versus wild type	*ALK/ROS1* fusion (+) versus wild type
No. (total 453)	170 (37.5)	41 (9.0)				
Age				<0.001 (*χ* ^2^ = 14.612)	0.471 (*χ* ^2^ = 0.660)	<0.001 (*χ* ^2^ = 19.920)
≤60	68 (40.0)	30 (73.2)	88 (36.1)			
>60	102 (60.0)	11 (26.8)	156 (63.9)			
Mean ± SD	61.85 ± 9.79	55.49 ± 12.62	62.97 ± 9.76	<0.001	0.254	<0.001
Range	27–86	30–85	28–87			
Gender				0.996	<0.001 (*χ* ^2^ = 61.934)	<0.001 (*χ* ^2^ = 28.152)
Male	83 (48.8)	20 (48.8)	207 (84.8)			
Female	87 (51.2)	21 (51.2)	37 (15.2)			
Smoking				0.162 (*χ* ^2^ = 1.955)	<0.001 (*χ* ^2^ = 59.315)	0.001 (*χ* ^2^ = 11.080)
No smokingc	130 (76.5)	27 (65.9)	93 (38.1)			
Smoking	40 (23.5)	14 (34.1)	151 (61.9)			
Histologic type				0.636 (*χ* ^2^ = 0.906)	<0.001 (*χ* ^2^ = 44.864)	0.002 (*χ* ^2^ = 12.318)
Adenocarcinoma	154 (90.6)	37 (90.2)	152 (62.3)			
Squamous	13 (7.6)	4 (9.8)	89 (36.5)			
Adenosquamous	3 (1.8)	0 (0)	3 (1.2)			
Disease stage				0.269 (*χ* ^2^ = 3.932)	0.002 (*χ* ^2^ = 14.642)	0.403 (*χ* ^2^ = 2.926)
I	16 (9.4)	4 (9.8)	13 (5.3)			
II	10 (5.9)	0 (0)	7 (2.9)			
III	20 (11.8)	8 (19.5)	62 (25.4)			
IV	124 (72.9)	29 (70.7)	162 (66.4)			
Lymphangitis	28 (16.5)	10 (24.4)	29 (11.9)	0.259 (*χ* ^2^ = 1.403)	0.194 (*χ* ^2^ = 1.774)	0.046 (*χ* ^2^ = 4.647)
Lymphadenopathy	119 (70.0)	29 (70.7)	185 (75.8)	0.927 (*χ* ^2^ = 0.008)	0.214 (*χ* ^2^ = 1.739)	0.558 (*χ* ^2^ = 0.486)
Emphysema	25 (14.7)	4 (9.8)	103 (42.2)	0.613 (*χ* ^2^ = 0.683)	<0.001 (*χ* ^2^ = 35.494)	<0.001 (*χ* ^2^ = 15.770)
Fibrosis	15 (8.8)	2 (4.9)	39 (16.0)	0.535 (*χ* ^2^ = 0.694)	0.038 (*χ* ^2^ = 4.529)	0.089 (*χ* ^2^ = 3.515)
Pleural effusion	71 (41.8)	15 (36.6)	91 (37.3)	0.598 (*χ* ^2^ = 0.367)	0.413 (*χ* ^2^ = 0.840)	0.931 (*χ* ^2^ = 0.008)

### 3.3 The association between *EGFR*, *ALK&ROS1* genes status and clinical characteristics

Compared with *EGFR*‐negative cases, the most were female (*χ* ^2^ = 45.938, *P* < 0.001), non‐smoking (*χ* ^2^ = 51.838, *P* < 0.001), and adenocarcinoma (*χ* ^2^ = 37.731, *P* < 0.001) patients in the *EGFR*‐positive group. In addition, there were significant differences in clinical stage (*χ* ^2^ = 14.554, *P* = 0.002) and proportion of patients with emphysema (*χ* ^2^ = 27.454, *P* < 0.001). There were no significant differences in age, lymphangitis, lymphadenopathy, fibrosis, and pleural effusion.

Compared with the *ALK&ROS1*‐negative group, the *ALK&ROS1*‐positive group was younger than the *ALK&ROS1*‐negative group (*χ* ^2^ = 19.805, *P* < 0.001). In the *ALK&ROS1*‐positive group, the most were female (*χ* ^2^ = 7.644, *P* = 0.008) patients, and proportion of patients with emphysema was lower (*χ* ^2^ = 8.202, *P* = 0.003) than the *ALK&ROS1*‐negative group. There were no significant differences in smoking history, histologic type, clinical stage, lymphangitis, lymphadenopathy, fibrosis, and pleural effusion between the *ALK&ROS1*‐negative group and the *ALK&ROS1*‐positive group (Table 3).

**TABLE 3:** Analysis of the relationship between *EGFR* and *ALK/ROS1* genes status and clinical characteristics

Characteristic	*EGFR* mutation			*ALK/ROS1* fusion		
	+	−	*P* value	+	−	*P* value
No. (total 453)	170 (37.5)	283 (62.5)		41 (9.1)	412 (90.9)	
Age			0.844 (*χ* ^2^ = 0.043)			<0.001 (*χ* ^2^ = 19.805)
≤60	68 (40.0)	116 (41.0)		30 (73.2)	154 (37.4)	
>60	102 (60.0)	167 (59.0)		11 (26.8)	258 (62.6)	
Gender			<0.001 (*χ* ^2^ = 45.938)			0.008 (*χ* ^2^ = 7.644)
Male	83 (48.8)	225 (79.5)		20 (48.8)	288 (69.9)	
Female	87 (51.2)	58 (20.5)		21 (51.2)	124 (30.1)	
Smoking			<0.001 (*χ* ^2^ = 51.838)			0.142 (*χ* ^2^ = 2.245)
No smoking	130 (76.5)	118 (41.7)		27 (65.9)	221 (53.6)	
Smoking	40 (23.5)	165 (58.3)		14 (34.1)	191 (46.4)	
Histologic type			<0.001 (*χ* ^2^ = 37.731)			0.063 (*χ* ^2^ = 5.525)
Adenocarcinoma	154 (90.6)	187 (66.1)		37 (90.2)	304 (73.8)	
Squamous	13 (7.6)	93 (32.9)		4 (9.8)	102 (24.8)	
Adenosquamous	3 (1.8)	3 (1.1)		0 (0)	6 (1.5)	
Disease stage			0.002 (*χ* ^2^ = 14.554)			0.554 (*χ* ^2^ = 2.090)
I	16 (9.4)	17 (6.0)		4 (9.8)	29 (7.0)	
II	10 (5.9)	7 (2.5)		0 (0)	17 (4.1)	
III	20 (11.8)	70 (24.7)		8 (19.5)	82 (19.9)	
IV	124 (72.9)	189 (66.8)		29 (70.7)	284 (68.9)	
Lymphangitis	28 (16.5)	39 (13.8)	0.495 (*χ* ^2^ = 0.610)	10 (24.4)	57 (13.8)	0.102 (*χ* ^2^ = 3.297)
Lymphadenopathy	119 (70.0)	212 (74.9)	0.275 (*χ* ^2^ = 1.302)	29 (70.7)	302 (73.3)	0.714 (*χ* ^2^ = 0.125)
Emphysema	25 (14.7)	107 (37.8)	<0.001 (*χ* ^2^ = 27.454)	4 (9.8)	128 (31.1)	0.003 (*χ* ^2^ = 8.202)
Fibrosis	15 (8.8)	41 (14.5)	0.079 (*χ* ^2^ = 3.145)	2 (4.9)	54 (13.1)	0.209 (*χ* ^2^ = 2.331)
Pleural effusion	71 (41.8)	105 (37.1)	0.370 (*χ* ^2^ = 0.972)	15 (36.6)	161 (39.1)	0.867 (*χ* ^2^ = 0.097)

### 3.4 The relationship between various types of mutations in the *EGFR* gene and clinical characteristics

Patients with mutations at 2 or more locations of the *EGFR* gene and with T790M resistance mutation were excluded from this analysis. Among the exon 19 deletion, L858R, L861Q, G719X mutations, and exon 20 insertion in *EGFR*, there were no significant differences in age and gender. The NSCLC patients with exon 19 deletion (73.3%) and L858R (85.0%) most were non‐smokers, whereas patients with L861Q (60.0%) most were smokers (Table 4). But the sample size of patients with L861Q, G719X mutations, and exon 20 insertion in our study is relatively small, and this result cannot represent the actual situation and we need a large sample size to analyze this problem.

**TABLE 4:** Analysis of the relationship between various types of mutations in the *EGFR* gene and clinical characteristics

Characteristic	Exon 19 deletion	L858R	L861Q	G719X	Exon 20 insertion	*P* value
No. (total 158)	90 (56.9)	60 (38.0)	5 (3.2)	1 (0.6)	2 (1.3)	
Age						0.058 (*χ* ^2^ = 9.145)
≤60	44 (48.9)	17 (28.3)	2 (40.0)	1 (100)	0 (0)	
>60	46 (51.1)	43 (71.7)	3 (60.0)	0 (0)	2 (100)	
Gender						0.368 (*χ* ^2^ = 4.289)
Male	46 (51.1)	25 (41.7)	4 (80.0)	0 (0)	1 (50.0)	
Female	44 (48.9)	35 (58.3)	1 (20.0)	1 (100.0)	1 (50.0)	
Smoking						0.102 (*χ* ^2^ = 7.725)
No smoking	66 (73.3)	51 (85.0)	2 (40.0)	1 (100)	1 (50.0)	
Smoking	24 (26.7)	9 (15.0)	3 (60.0)	0 (0)	1 (50.0)	
Lymphangitis	15 (16.7)	7 (11.7)	1 (20.0)	1 (100)	1 (50.0)	0.093 (*χ* ^2^ = 7.966)
Lymphadenopathy	56 (62.2)	46 (76.7)	4 (80.0)	1 (100)	1 (50.0)	0.334 (*χ* ^2^ = 4.569)
Emphysema	18 (20.0)	6 (10.0)	0 (0.0)	0 (0)	0 (0)	0.366 (*χ* ^2^ = 4.304)
Fibrosis	7 (7.8)	6 (10.0)	1 (20.0)	0 (0)	0 (0)	0.864 (*χ* ^2^ = 1.287)
Pleural effusion	38 (42.2)	26 (43.3)	3 (60.0)	1 (100)	1 (50.0)	0.746 (*χ* ^2^ = 1.944)

### 3.5 Logistic regression analysis of variables associated with *EGFR* mutations and *ALK/ROS1* gene fusions

Logistic regression analysis was performed to determine independent variables associated with *EGFR* mutations and *ALK/ROS1* gene fusions. The results indicated that female (adjusted odds ratio [OR] 1.834, 95% confidence interval [CI] 1.069–3.144, *P* = 0.028), non‐smoking (adjusted OR 2.504, 95% CI 1.456–4.306, *P* = 0.001), lung adenocarcinoma (adjusted OR 4.512, 95% CI 2.465–8.260, *P* < 0.001), stage III–IV (adjusted OR 2.232, 95% CI 1.066–4.676, *P* = 0.033), and no symptoms of emphysema (adjusted OR 2.139, 95% CI 1.221–3.747, *P* = 0.008) were independent variables associated with *EGFR* mutations (Table 5). Young (adjusted OR 3.947, 95% CI 1.873–8.314, *P* < 0.001) and lung adenocarcinoma (adjusted OR 2.950, 95% CI 0.998–8.719, *P* = 0.050) were independent variables associated with *ALK/ROS1* gene fusions (Table 5).

**TABLE 5:** Logistic regression analysis of variables associated with *EGFR* mutations and *ALK/ROS1* gene fusions

Variables	*EGFR* mutations				*ALK/ROS1* gene fusions			
	Unadjusted values		Adjusted values		Unadjusted values		Adjusted values	
	*P* value	OR (95% CI)	*P* value	Adjusted OR (95% CI)	*P* value	OR (95% CI)	*P* value	Adjusted OR (95% CI)
Age (≤60/>60)	0.836	0.960 (0.651–1.414)	0.083	0.672 (0.428–1.054)	<0.001	4.569 (2.226–9.378)	<0.001	3.947 (1.873–8.314)
Gender (female/male)	<0.001	4.066 (2.680–6.169)	0.028	1.834 (1.069–3.144)	0.007	2.439 (1.276–4.660)	0.153	1.923 (0.784–4.714)
Smoking (no/yes)	<0.001	4.544 (2.968–6.958)	0.001	2.504 (1.456–4.306)	0.137	1.667 (0.850–3.270)	0.489	0.718 (0.281–1.835)
Histologic type (adenocarcinoma/non‐adenocarcinoma)	<0.001	4.941 (2.793–8.743)	<0.001	4.512 (2.465–8.260)	0.027	3.286 (1.145–9.435)	0.050	2.950 (0.998–8.719)
Disease stage (III–IV/I–II)	0.027	1.948 (1.079–3.519)	0.033	2.232 (1.066–4.676)	0.784	0.860 (0.293–2.523)	0.437	0.606 (0.171–2.143)
Lymphangitis (no/yes)	0.435	0.811 (0.478–1.374)	0.838	0.938 (0.506–1.738)	0.074	0.498 (0.231–1.070)	0.340	0.661 (0.282–1.548)
Lymphadenopathy (no/yes)	0.254	1.280 (0.837–1.956)	0.988	0.996 (0.589–1.683)	0.724	1.136 (0.560–2.304)	0.281	1.580 (0.688–3.625)
Emphysema (no/yes)	<0.001	3.526 (2.165–5.743)	0.008	2.139 (1.221–3.747)	0.008	4.169 (1.455–11.943)	0.087	2.696 (0.866–8.389)
Fibrosis (no/yes)	0.079	1.751 (0.937–3.270)	0.378	1.386 (0.671–2.864)	0.145	2.941 (0.690–12.533)	0.405	1.906 (0.418–8.692)
Pleural effusion (no/yes)	0.325	0.823 (0.558–1.213)	0.661	0.903 (0.573–1.425)	0.755	1.112 (0.571–2.163)	0.460	1.314 (0.636–2.714)

Abbreviations: CI, confidence interval; OR, odds ratio.

## 4 DISCUSSION

The number of lung cancer patients and death cases in China has ranked first among all malignant tumors.^3^, ^19^, ^20^ In the past decade, as the development of tumor molecular diagnosis and the continuous discovery of targeted drugs, the treatment of NSCLC has entered an era of individualized molecular targeted therapy. With the discovery of therapeutic targets EGFR, ALK, and ROS1 and the advent of corresponding targeted drugs, targeted therapy has become a very effective way to treat NSCLC clinically.^21^ Studies have shown that NSCLC patients with *EGFR* sensitive mutations and *ALK&ROS1* gene fusions can benefit from corresponding targeted therapy.^10^, ^22^, ^23^ Therefore, detection of *EGFR*, *ALK&ROS1* gene mutations have important clinical significance in screening patients suitable for targeted therapy. In the present study, the clinical characteristics of NSCLC with *ALK&ROS1* gene rearrangement and *EGFR* mutations were investigated. Distinguishing the clinical characteristics of different molecular subtypes will be beneficial to the diagnosis and treatment of lung cancer.

EGFR is a transmembrane receptor tyrosine kinase, and the activation or phosphorylation of this region is of great significance for the signaling of proliferation and growth of cancer cells. *EGFR* mutation mainly includes four types: deletion mutations in exon 19, point mutations in exon 21, point mutations in exon 18, and insertion mutations in exon 20, among which exon 19 deletion mutation and exon 21 L858R are the most common mutations sensitive to EGFR‐TKI therapy.^24^ In concordance with previous reports, *EGFR* mutations were mainly 19 exon deletion mutations and exon 21 L858R mutation in 453 NSCLC patients of this study. A number of researches have shown that the incidence of *EGFR* mutation is higher in women, non‐smokers, and adenocarcinoma.^25^, ^26^ This study found that the incidence of *EGFR* mutation in women, adenocarcinoma, and non‐smokers is significantly higher than that in men, squamous cell carcinoma, and smokers, and the differences between these groups are statistically significant.

Since Soda *et al*.^27^ first discovered a new *EML4‐ALK* gene fusion in NSCLC patients in 2007, many scholars have conducted studies on it. Regarding the mutation rate of *EML4‐ALK* in NSCLC, different literatures reported slight differences. Domestic and foreign research data showed that the incidence of *ALK* gene fusion in NSCLC patients was 3%–7%.^28^, ^29^, ^30^ The positive rate of *ROS1* gene fusion in NSCLC was 1.0%–3.4%,^31^ and the clinical characteristics of *ALK* and *ROS1* gene fusion lung cancer were also very similar. The results of this study showed that the positive rate of *ALK* and *ROS1* genes fusions was 9.1%, and the incidence of *ALK* and *ROS1* gene fusions were relatively high in female patients and those less than 60 years old. *ALK* gene fusions were identified in 32 cases (33/453, 7.3%) and *ROS1* gene fusions were identified in 9 cases (9/453, 2.0%). Our work also confirms the low incidence of the *ALK&ROS1* fusion among unselected NSCLC patients.

In this study, compared with non‐*ALK&ROS1/EGFR* mutations in NSCLC patients, patients with *EGFR* mutation had a lower incidence of pulmonary emphysema. And it reflected that most of NSCLC patients with *EGFR* mutation had a history of non‐smoking. A plausible reason for this is that *EGFR* mutation status have a stronger association with non‐emphysema status. Among the deletions in exon 19, L858R, L861Q, G719X, S768I mutations, and insertions in exon 20 of *EGFR*, there were no significant differences in age and gender. The NSCLC patients with deletions in exon 19 (74.7%) and L858R (86.4%) most were non‐smokers, whereas patients with L861Q (62.5%) and G719X (66.7%) most were smokers. But the sample size of patients with L861Q, G719X, S768I mutations, and insertions in exon 20 in our study is relatively small, and this result cannot represent the actual situation and we need a large sample size to analyze this problem.

According to reports, the incidence of *ALK* gene rearrangement in NSCLC patients is about 3%–7%,^10^ whereas the incidence of *EGFR* mutation is 40%–80%^32^; the sample size of patients with *ALK&ROS1* gene rearrangement is small in our study. This is one of the limitations in this study. The purpose of this study was to evaluate the clinical characteristics of NSCLC patients to distinguish between *ALK&ROS1* gene rearrangement, *EGFR* mutation, and non‐*ALK&ROS1/EGFR* mutation. These results may assist clinicians to assess the NSCLC patients with these genetic abnormalities.

## 5 CONCLUSIONS

In conclusion, this study suggests that *EGFR* mutations were more likely to occur in non‐smoking, stage III–IV, and female patients with lung adenocarcinoma, whereas *ALK&ROS1* gene rearrangements were more likely to occur in young patients with lung adenocarcinoma. Emphysema was less common in patients with *EGFR* mutations.

## AUTHOR CONTRIBUTIONS

Qinghua Liu and Heming Wu designed the study. Heming Wu and Zhikang Yu performed the experiments. Qinghua Liu recruited subjects and collected clinical data. Qingyan Huang and Zhikang Yu helped to analyze the data. Heming Wu prepared the manuscript. All authors were responsible for critical revisions, and all authors read and approved the final version of this work.

## CONFLICT OF INTEREST

The authors declare that there is no conflict of interest.

## ETHICS STATEMENT

All protocols were approved by the Human Ethics Committees of Meizhou People's Hospital, Meizhou Academy of Medical Sciences.

## Data Availability

The data that support the findings of this study are available from the corresponding author upon reasonable request.
